# 

**Published:** 1906-10-15

**Authors:** 


### ANNOUNCEMENTS.

DEAD.—Gerald G. McKellops, a son of the late Dr. J. H. McKellops, of St. Louis, died in Cincinnati, August 18, 1906.

It was the earnest desire of his father that Gerald should become a dentist. He sent him to Ann Arbor, where he attended the dental college for two years, but made so little progress that he dropped the course and went into business in Cincinnati, where he died under most unfortunate circumstances, due to his having become the slave of the drug habit.

OFFICERS OF THE NATIONAL DENTAL ASSOCIATION.—At the tenth annual session of the National Dental Association, held at Atlanta, Georgia, Sept. 18-21, 1906, the following officers were elected for the ensuing year.

President, A. H. Peck, Chicago, Ill.; Vice-President, for West, D. J. McMillen, Kansas City; Vice-President for East, George E. Hunt, Indianapolis, Ind.; Vice-President for South, George S. Vann, Gadsden, Ala.; Recodring Sec'y., Chas. B. Butler, Buffalo; Corresponding Sec'y., Burton Lee Thorpe, St. Louis; Treasurer, A. R. Melendy, Knoxville, Tenn.; Executive Council, N. J. Burkhart, Batavia, N. Y., J. Y. Crawford, Nashville, Tenn., Chas McManus, Hartford, Conn., F. O. Hetrick, Ottawa, Kansas, B. Holly Smith, Baltimore, Md.; Executive Committee.—C. M. Work, Ottuwa, Ia., V. H. Jackson, New York City, N. Y., Thomas P. Hinman, Atlanta, Ga.

---

SOMNOFORM DEATH.—Mrs. Grace Herbig, an apparently heatlhy woman, 28 years of age, died in the office of the Hays Dental Co., at Rockport, Ills., September 12th.

The somnoform was administered and the tooth to be extracted broke so that one of the roots remained in the jaw. After half an hour another administration was made and the root removed. The patient did not recover promptly and the dentist administered whisky and injected nitro-glycerine, but death resulted in spite of all the usual efforts of the dentists and a physician, who was called. The coroners jury was unable to determine the cause of the death.

---

OFFICERS SOUTHERN NEBRASKA DENTAL SOCIETY.—At a meeting held August 30th, at Red Cloud, the following officers were elected for the ensuing year: President, Dr. Frank Nelson, of Superior; Vice-President, Dr. J. N. Prime, Oxford; Secretary and Treasurer, Dr. W. A. McHenry, Nelson.

The next meeting will be held November 14, 1906, at Superior.

---

OHIO STATE BOARD OF DENTAL EXAMINERS.—The regular semi-annual meeting of this Board will be held at Columbus, Ohio, commencing November 27, 1906. Only graduates are eligible to examination. All candidates must file an application with the Secretary on or before November 17th accompanied by the fee which is twenty dollars.

For full particulars address, Dr. H. C. Brown, Secretary, 185 East State Street, Columbus, Ohio.

—✦—

MICHIGAN STATE BOARD OF EXAMINERS.—Will meet at the Detroit College of Medicine in Detroit, October 24, 1906.

For full particulars address, Dr. A. L. LeGro, Sercetary, Three Rivers, Mich.

—✦—

ILLINOIS STATE BOARD OF EXAMINERS.—Will meet at the Chicago College of Dental Surgery, in Chicago, Monday November 12, 1906,

Persons who have been legal practitioners in other states for five years, or graduates of reputable colleges, or graduates of reputable medical colleges, with dental acquirements, may take the examination.

Credentials must be furnished the Secretary five days previous to the examination. Address Dr. Jas. G. Reed, 1204 Trude Building, Chicago, Ill.

—✦—

OFFICERS NORTHERN OHIO DENTAL ASSOCIATION.—At the annual meeting in Cleveland, June 5th, the following officers were elected for the ensuing year; President, Dr. J. R. Owens, of Cleveland; Vice-President, Dr. C. A. Allen, of Toledo; Corresponding Secretary, Dr. D. H. Ziegler, of Cleveland; Recording Secretary, Dr. J. K. Douglas, Sandusky.

—✦—

HARVARD DENTAL ALUMNI ASSOCIATION.—At its 35th annual meeting this organization elected the following officer for the ensuing year. President, Arthur W. Eldred, Worcester, Mass.; Vice-President, Harvey W. Hardy, Boston; Secretary, W. E. Boardman, 184 Boylston Street, Boston.

